# Cisplatin dose rate as a risk factor for nephrotoxicity in children.

**DOI:** 10.1038/bjc.1998.276

**Published:** 1998-05

**Authors:** R. Skinner, A. D. Pearson, M. W. English, L. Price, R. A. Wyllie, M. G. Coulthard, A. W. Craft

**Affiliations:** Sir James Spence Institute of Child Health, University of Newcastle upon Tyne, Royal Victoria Infirmary, UK.

## Abstract

The purpose of the study was to evaluate the incidence, risk factors and changes in severity with time of cisplatin nephrotoxicity in children. A total of 35 children underwent measurement of glomerular filtration rate (GFR) and tubular function after completion of cisplatin chemotherapy. No child received ifosfamide. A clinically relevant 'nephrotoxicity score' was derived from GFR and serum magnesium. Follow-up studies were performed in 16 children at 1 year and in 15 at 2 years after cisplatin. Considerable interpatient variability in nephrotoxicity was observed. Treatment was modified in three patients because of nephrotoxicity. GFR was low in 18 out of 31 patients. Proximal nephron toxicity caused hypomagnesaemia in ten patients and hypocalcaemia in five patients. Elevated urinary N-acetylglucosaminidase excretion was seen in 22 out of 30 children, indicating subclinical tubular toxicity. Nephrotoxicity was less severe in children who received cisplatin courses at a dose rate of 40 mg m(-2) day(-1) than in those who received higher dose rates (P < 0.005), but there was no correlation with total dose received. Follow-up studies revealed partial recovery of GFR (P < 0.05). Glomerular and proximal nephron toxicity are common in children treated with cisplatin, and more severe at higher dose rates. Despite partial recovery of GFR, the long-term outcome of nephrotoxicity remains unknown and careful monitoring of chronic toxicity is necessary.


British Journal of Cancer (1998) 77(10), 1677–1682
© 1998 Cancer Research Campaign

### Cisplatin dose rate as a risk factor for nephrotoxicity in children

**R Skinner, ADJ Pearson, MW English, L Price, RA Wyllie, MG Coulthard and AW Craft**

Sir James Spence Institute of Child Health, University of Newcastle upon Tyne, Royal Victoria Infirmary, Queen Victoria Road, Newcastle upon Tyne NE1 4LP, UK

**Summary** The purpose of the study was to evaluate the incidence, risk factors and changes in severity with time of cisplatin nephrotoxicity in children. A total of 35 children underwent measurement of glomerular filtration rate (GFR) and tubular function after completion of cisplatin chemotherapy. No child received ifosfamide. A clinically relevant 'nephrotoxicity score' was derived from GFR and serum magnesium. Follow-up studies were performed in 16 children at 1 year and in 15 at 2 years after cisplatin. Considerable interpatient variability in nephrotoxicity was observed. Treatment was modified in three patients because of nephrotoxicity. GFR was low in 18 out of 31 patients. Proximal nephron toxicity caused hypomagnesaemia in ten patients and hypocalcaemia in five patients. Elevated urinary *N*-acetylglucosaminidase excretion was seen in 22 out of 30 children, indicating subclinical tubular toxicity. Nephrotoxicity was less severe in children who received cisplatin courses at a dose rate of 40 mg m$^{-2}$ day$^{-1}$ than in those who received higher dose rates ($P < 0.005$), but there was no correlation with total dose received. Follow-up studies revealed partial recovery of GFR ($P < 0.05$). Glomerular and proximal nephron toxicity are common in children treated with cisplatin, and more severe at higher dose rates. Despite partial recovery of GFR, the long-term outcome of nephrotoxicity remains unknown and careful monitoring of chronic toxicity is necessary.

**Keywords:** cisplatin; nephrotoxicity; children

The use of combination chemotherapy has led to dramatic improvements in the survival rates from many childhood malignancies during the last three decades. However, cytotoxic drugs may also cause severe and chronic side-effects, leading to permanent ill-health, disability or even premature death (Morris Jones and Craft, 1990). The development of effective strategies to prevent these adverse events depends on careful evaluation of toxicity both during and after treatment.

In view of its established efficacy, cisplatin has retained an important role in the treatment of several childhood solid tumours, including neuroblastoma, osteosarcoma, hepatic and brain tumours, despite the increasing use of its apparently less nephrotoxic analogue carboplatin. Indeed, recent evidence has suggested that cisplatin may be more active than carboplatin in some germ cell tumours in adolescents and adults (Bajorin et al, 1993). However, even with the use of hyperhydration and other protective measures, nephrotoxicity is still a common and potentially serious adverse effect of cisplatin in both adults (Madias and Harrington, 1978; Schilsky et al, 1982; Daugaard and Abildgaard, 1989) and children (Womer et al, 1985; Brock et al, 1991). Despite numerous publications concerning cisplatin nephrotoxicity in adults, there is less information and no clear consensus on the importance of patient- and treatment-related risk factors for the development of nephrotoxicity in children. Furthermore, as there are few data concerning the long-term outcome of cisplatin nephrotoxicity in children (Brock et al, 1991), especially that of tubular toxicity, concern persists over the possibility that significant chronic renal damage may become evident only in adult life.

*Received 10 June 1997*
*Revised 27 August 1997*
*Accepted 26 September 1997*

*Correspondence to:* R Skinner

Therefore, this study was designed to investigate these issues. The aims were to evaluate first, the incidence, nature and severity of glomerular and renal tubular toxicity in children treated with cisplatin; second, the relevance of patient- and treatment-related risk factors in the development of such toxicity; and finally, the changes in renal function with time after completion of cisplatin.

### METHODS

### Patients and treatment

Between August 1988 and August 1991, 35 children and adolescents resident in the Northern Health Region of England received cisplatin chemotherapy and were followed up after treatment at the Regional Paediatric Oncology Unit in Newcastle upon Tyne. No child died from cisplatin nephrotoxicity. The mean age at diagnosis was 5.7 (range 0.6–17.8) years. A total of 16 children had neuroblastoma, seven had a brain tumour, five each had osteosarcoma or had a germ cell tumour, and two had a hepatic tumour. At diagnosis, all children had a normal serum creatinine concentration. Glomerular filtration rate (GFR) was measured from the plasma clearance of $^{51}$Cr-labelled ethylenediaminetetraacetic acid ([$^{51}$Cr]EDTA) before cisplatin treatment in 30 children, and was normal (≥87 ml min$^{-1}$ 1.73 m$^{-2}$) in 28 but slightly reduced in the other two. One child with partial urinary tract obstruction at diagnosis due to tumour had a GFR of 72 ml min$^{-1}$ 1.73 m$^{-2}$. The other child had a marginally low GFR of 81 ml min$^{-1}$ 1.73 m$^{-2}$ at diagnosis, without obvious cause. All children had normal renal tubular function as defined by normal serum electrolyte, calcium and magnesium concentrations, and normal urinalysis. No child had renal invasion by tumour nor urinary tract obstruction. The study protocol received local institutional ethics committee approval. Informed verbal consent was obtained from the patients and/or parents.

After completion of chemotherapy, all 35 patients underwent an initial 'baseline' study at a median of 1 (1–21) month after the last cisplatin treatment (within 3 months in 23 patients). During the study period (i.e. August 1988–91), all patients surviving to 1 or 2 years after completion of cisplatin were restudied. A total of 16 of the 35 children underwent a '1-year post-treatment' study (1-year study) at a median of 14 (11–19) months post-cisplatin, and 15 a '2-year post-treatment' study (2-year study) at a median of 25 (20–31) months post-cisplatin. The 1-year study was performed at a median of 11 (range 4–14) months, and the 2-year study 20 (range 7–27) months after the baseline study.

The mean total dose of cisplatin received was 568 (291–750) mg $m^{-2}$, given over a mean of 5.0 (2–8) courses. Several schedules of cisplatin were used, all using intravenous hydration at 3 l $m^{-2}$ $day^{-1}$. The cisplatin dose per course ranged from 60 to 200 mg $m^{-2}$, given as a continuous infusion (40–100 mg $m^{-2}$ $day^{-1}$) over 1–5 days, except in the children with germ cell tumours (20-min infusion). All high dose (200 mg $m^{-2}$) cisplatin courses were given over 5 days (i.e. 40 mg $m^{-2}$ $day^{-1}$). The treatment protocols were divided into two groups according to the rate at which cisplatin was delivered during each treatment course (i.e. cisplatin dose rate). Those protocols for neuroblastoma and brain tumours were characterized by a 'low' cisplatin dose rate (40 mg $m^{-2}$ $day^{-1}$, $n = 23$), and those for osteosarcoma, germ cell or hepatic tumours by a 'high' dose rate [>40–120 mg $m^{-2}$ (mean 96.3, standard error 4.3), $n = 12$]. Treatment courses were repeated every 3 weeks, except in children with neuroblastoma who received a rapid scheduling protocol with courses of 80 and 200 mg $m^{-2}$ at 10–20 day intervals.

Other chemotherapy included actinomycin D, bleomycin, carboplatin, cyclophosphamide, doxorubicin, etoposide, melphalan and vincristine. The carboplatin (1 g $m^{-2}$) and melphalan (180 mg $m^{-2}$) were given in single doses as part of high-dose chemotherapy before autologous bone marrow transplantation in seven children with neuroblastoma. No child received ifosfamide or radiotherapy to a treatment field that included the kidneys. Potentially nephrotoxic supportive treatment (intravenous aminoglycosides, vancomycin, acyclovir or amphotericin B) was given to 28 of the children for between 6 and 154 days (mean 35.3 days for whole group).

### Investigations

The clinical consequences of nephrotoxicity were recorded, in particular tetany. Renal function was assessed using a standardized protocol (Skinner et al, 1991).

### *Glomerular function*

Serum creatinine concentration and GFR (using the [$^{51}$Cr]EDTA plasma clearance method) were measured.

### *Proximal nephron function*

Concentrations of electrolytes, creatinine, calcium, magnesium, phosphate and glucose were measured in corresponding blood and urine samples, with calculation of the fractional excretions of sodium, potassium, calcium, magnesium, phosphate and glucose, and of the renal tubular threshold for phosphate ($Tm_p$/GFR) (Skinner et al, 1991). The fractional excretion of a substance is the percentage of the filtered load at the glomerulus that is subsequently excreted in the urine; an abnormally high fractional excretion indicates reduced tubular reabsorption. The majority of reabsorption of the above molecules occurs in the proximal nephron. Except with glucose, a fractional excretion was considered abnormal only when it was elevated in the presence of a reduced blood concentration. The $Tm_p$/GFR provides a measure of tubular phosphate reabsorption, being reduced in the presence of impaired reabsorption.

In addition, the urine excretion of a low-molecular-weight protein, either $\beta_2$-microglobulin ($\beta_2$-M) or retinol-binding protein (RBP), was measured and expressed as a ratio to the urine creatinine concentration in the same sample. Increased urine excretion indicates reduced proximal nephron reabsorption.

### *Distal nephron function*

Serum bicarbonate concentration and early morning urine pH were measured to evaluate both proximal and distal nephron regulation of acid–base balance. Early morning urine osmolality was determined as a measure of urinary concentration. Although impaired urinary concentration may result from either proximal or distal nephron dysfunction, severe impairment is more likely to be observed in distal nephron toxicity (Skinner et al, 1991).

### *General aspects of renal function*

The urinary concentrations of two renal tubular enzymes [alanine aminopeptidase (AAP) and *N*-acetylglucosaminidase (NAG)] and of total protein were measured and expressed as ratios to the urine creatinine concentration. Increased urine excretion of AAP and NAG implies tubular damage. Blood pressure was also measured.

### *Nephrotoxicity grading*

Nephrotoxicity grading was performed using a system that grades (on a 0–4 scale) GFR and serum magnesium concentration, with summation to give a 'nephrotoxicity score', potentially ranging from 0 to 8, based on the common clinically relevant aspects of cisplatin nephrotoxicity (Table 1).

### Reference ranges (Table 2)

Reference ranges for serum biochemistry and fractional excretions were obtained from investigation of 105 otherwise healthy children and adolescents (aged 0.1–16.6 years, 27 male children) attending hospital for investigation of a proven urinary tract infection (treated at least 1 month previously), in whom clinical examination, renal and urinary tract investigations and imaging proved to be normal. Age-related reference ranges for RBP, pH, osmolality, AAP and NAG were derived from early-morning urine samples in 322 normal schoolchildren and students (aged 3.9–18.7 years, 170 male patients) with no personal or family history of renal disease. Previously outlined reference ranges (Skinner et al, 1991) were used for GFR, $\beta_2$-M, and age- or sex-dependent biochemical variables (serum creatinine, bicarbonate and magnesium).

### Statistical analysis

Multiple linear regression analysis was used to evaluate age at start of treatment, total cisplatin dose, cisplatin dose rate, time since completion of cisplatin (in months), sex and duration (in days) of other potentially nephrotoxic drugs as predictors for clinically relevant nephrotoxicity measured by GFR, serum magnesium, fractional excretion of magnesium and nephrotoxicity score. Initially, all predictor variables were included simultaneously in multiple regression to determine which had a significant influence

© Cancer Research Campaign 1998

**Table 1** Grading of cisplatin nephrotoxicity in children

| Nephrotoxicity grade | GFR | Mg | |
|---|---|---|---|
| | | <2 years | ≥2 years |
| 0 | ≥90 | ≥0.75 | ≥0.70 |
| 1 | 60–89 | 0.60–0.74 | 0.55–0.69 |
| 2 | 40–59 | 0.50–0.59 | 0.45–0.54 |
| 3 | 20–39 | No symptoms, but 0.40–0.49 | 0.35–0.44 |
| 4 | <20 | Tetany or convulsion or <0.40 | <0.35 |

Total score ($N_s$) (i.e. sum of GFR + Mg)
0 No nephrotoxicity
1 Mild nephrotoxicity
2–3 Moderate nephrotoxicity
≥4 Severe nephrotoxicity

GFR, glomerular filtration rate (ml $min^{-1}$ 1.73 $m^{-2}$); Mg, serum magnesium concentration (mmol $1^{-1}$). Tetany is defined by clinical symptoms/signs (carpopedal spasm, Chvostek's sign, Trousseau's sign) with biochemistry (moderate or severe hypomagnesaemia – <0.60 at <2 years of age, <0.55 at ≥2 years). Hypocalcaemia may also cause tetany. If hypomagnesaemia and hypocalcaemia co-exist in the presence of tetany, assume that hypomagnesaemia is the primary cause unless there are good reasons not to do so, and grade appropriately. A score of 4 in an individual aspect of grading (e.g. GFR) constitutes severe toxicity in that aspect.

**Table 2** Renal function at the baseline study

| | **Mean (s.d.)** | **Range** | **Number (%) of abnormal results** | **Reference range** |
|---|---|---|---|---|
| Glomerular function | | | | |
| GFR (ml $min^{-1}$ 1.73 $m^{-2}$) | 87.2 (31.5) | 18–151 | 18/31 (58) | 87–174 |
| Proximal nephron function | | | | |
| Serum magnesium (mmol $l^{-1}$) | 0.75 (0.12) | 0.44–0.95 | 10/35 (29) | 0.70–1.00[a] |
| Serum sodium (mmol $l^{-1}$) | 139.2 (2.6) | 134–147 | 9/35 (26) | 137–144 |
| Serum calcium (mmol $l^{-1}$) | 2.44 (0.12) | 2.12–2.69 | 5/35 (14) | 2.30–2.63 |
| Serum chloride (mmol $l^{-1}$) | 105.5 (3.6) | 95–110 | 2.35 (6) | 100–109 |
| Urine $\beta_2$-M (mg mmol $creatinine^{-1}$) | 0.16 (0.38) | 0.006–1.30 | 10/11 (91) | <0.01 |
| FEglucose (%) | 0.2 (0.3) | 0.0–1.5 | 18/25 (72) | ≤0.05 |
| Urine RBP (μg mmol $creatine^{-1}$) | 1326 (2252) | 7–6000 | 5/7 (71) | ≤9.5–≤37.7[a] |
| FE magnesium (%) | 4.6 (3.1) | 1.1–11.2 | 2/29 (7) | 1.1–9.1 |
| Distal nephron function | | | | |
| EMUpH | 5.9 (0.9) | 4.7–8.0 | 21/30[b] (70) | ≤5.4 |
| EMUO (mOsm $kg^{-1}$)) | 646 (215) | 291–1064 | 13/29[b] (45) | ≥600 |
| General aspects | | | | |
| Urine NAG (U mmol $creatinine^{-1}$) | 0.98 (0.90) | 0.08–3.72 | 22/30 (73) | ≤0.34 |
| Urine protein[a] (mg mmol $creatine^{-1}$) | 43.5 (80.5) | 1.1–416.7 | 18/30 (60) | ≤8.2–≤19.3[a] |
| Urine AAP (U mmol $creatinine^{-1}$) | 5.63 (9.43) | 0.00–47.14 | 15/27 (56) | ≤1.7–≤2.5[a] |

s.d., standard deviation; FE, fractional excretion; EMUpH, early morning urine pH; EMUO, early morning urine osmolality. [a] Denotes age-related reference range. [b]The number of results not *proven* to be normal is indicated.

upon the indicators of nephrotoxicity. Only the cisplatin dose rate was found to be significant. Therefore, unpaired *t*-tests and Fisher's exact test were performed to compare the severity and frequency of toxicity, as well as total cisplatin dose and the duration of other potentially nephrotoxic treatment, in the 'low' and 'high' dose rate groups. Changes with time were assessed by paired *t*-tests.

### RESULTS

### Clinical consequences of nephrotoxicity

Cisplatin was discontinued early in two children who developed acute renal failure after three courses of cisplatin. One required peritoneal dialysis for a week. Both patients had other potential risk factors, notably very recent or concurrent exposure to other

© Cancer Research Campaign 1998

Table 3 Cisplatin dose rate and nephrotoxicity

| Measure of nephrotoxicity | 'Low' dose rate ($n$ = 21–23) | 'High' dose rate ($n$ = 10–12) | $P$ |
|---|---|---|---|
| Total cisplatin dose (mg m$^{-2}$) | 587 (32) | 532 (35) | 0.28 |
| Duration of other potentially nephrotoxic treatment (days) | 40.5 (8.1) | 26.1 (8.0) | 0.26 |
| GFR (ml min$^{-1}$ 1.73 m$^{-2}$) | 98.4 (5.4) | 63.7 (10.0) | 0.0025 |
| Serum magnesium (mmol l$^{-1}$) | 0.79 (0.02) | 0.66 (0.04) | 0.0007 |
| Nephrotoxicity score | 0.71 (0.16) | 2.40 (0.43) | 0.0001 |

Results quoted as mean (standard error); 'Low' dose rate, 40 mg m$^{-2}$ day$^{-1}$; 'High' dose rate, >40–120 mg m$^{-2}$ day$^{-1}$.

potentially nephrotoxic drugs (aminoglycosides and amphotericin B). Renal function improved in only one of these children; the other patient and a further child (given five courses of cisplatin) subsequently developed asymptomatic chronic renal failure (GFR <30 ml min$^{-1}$ 1.73 m$^{-2}$). The choice of subsequent chemotherapy at the time of relapse was limited by the renal failure in the last child. No patient developed tetany and none received long-term oral magnesium supplementation after completion of treatment.

### Renal function

The most important results (referring to the baseline study only) are summarized below (Table 2). Inadequate blood or urine samples (too small to permit biochemical analysis) and unsuccessful GFR measurements (failure to achieve complete intravenous injection of the radioisotope) were considered inevaluable.

### *Glomerular function*

Although GFR was low (range 18–86 ml min$^{-1}$ 1.73 m$^{-2}$) in 18 (58%) of 31 evaluable children, the serum creatinine concentration was elevated in only seven of these. The two children with low GFRs before cisplatin treatment did not demonstrate noticeable glomerular toxicity – the GFR increased from 72 (pre-cisplatin) to 79 ml min$^{-1}$ 1.73 m$^{-2}$ (1 year post treatment, GFR inevaluable at the end of treatment study) in one, and fell from 81 (pre-cisplatin) to 74 ml min$^{-1}$ 1.73 m$^{-2}$ (end of treatment), before rising to 94 and 97 ml min$^{-1}$ 1.73 m$^{-2}$ (1 and 2 years post treatment) in the other.

### *Proximal tubular function*

Ten (29%) children were hypomagnesaemic (serum magnesium ranging from 0.44 to 0.67 mmol l$^{-1}$). The two children with low GFRs before cisplatin had normal serum magnesium concentrations on all occasions of study. The fractional excretion of magnesium was measured in nine of the ten hypomagnesaemic children, being high in two and in the upper half of the reference range (i.e. between 5.1% and 9.1%) in two. The serum concentrations of sodium, chloride and calcium were decreased slightly in 26%, 6% and 14%, respectively, of children. The fractional excretion of glucose was increased in 18 (72%) of 25 evaluable children. The plasma glucose concentration ($n$ = 35) and the fractional excretions of sodium ($n$ = 21) and calcium ($n$ = 28) were normal in all evaluable children. No child had an abnormal serum concentration or fractional excretion of potassium or phosphate, or $Tm_p$/GFR.

The urinary $\beta_2$-M/creatinine and RBP/creatinine ratios were elevated in 10 of 11, and in five of seven evaluable children respectively.

### *Distal tubular function*

The early morning urine pH was ≤5.4 in only 9 (30%) of 30 evaluable children, and osmolality ≥600 mOsm kg$^{-1}$ in only 16 (55%) of 29. However, no child had polydipsia, polyuria or a reduced serum bicarbonate concentration.

### *General aspects of renal function*

The urine AAP/creatinine, NAG/creatinine and total protein/creatinine ratios were elevated in 15 (56%) of 27, 22 (73%) of 30, and 18 (60%) of 30 evaluable children respectively. Systolic blood pressure was elevated without other apparent cause in one patient.

### *Grading scores*

One patient had severe (grade 4) glomerular toxicity, but no child had grade 4 tubular toxicity. The mean (range) GFR score was 0.84 (0–4), serum magnesium score 0.40 (0–3), and overall nephrotoxicity score 1.26 (0–5). The overall score was evaluable in 31 children – no toxicity was seen in ten (32%) patients, but ten had mild (i.e. nephrotoxicity score = 1), ten moderate (score = 2–3) and one (3%) severe toxicity (score ≥4).

### Patient- and treatment-related risk factors

Multiple regression analysis demonstrated that only the cisplatin dose rate exerted a significant ($P$ < 0.05) effect on the measures of nephrotoxicity evaluated in this study. Those children treated with the 'low' cisplatin dose rate had higher GFRs and serum magnesium concentrations, and lower nephrotoxicity scores than those treated with higher dose rates (all $P$ < 0.005). There was no significant difference between the two groups in total cisplatin dose received or the duration of other potentially nephrotoxic treatment (Table 3). Although total cisplatin dose did not have an independent effect on toxicity, the only four children with serum magnesium concentrations <0.60 mmol l$^{-1}$ had all received doses >500 mg m$^{-2}$. Only 3 of 21 evaluable 'low' cisplatin dose rate, but eight of ten 'high' dose rate patients, suffered moderate or severe toxicity (nephrotoxicity score ≥2) ($P$ = 0.0007).

### Changes in the severity of nephrotoxicity with time

Using paired data, the GFR was significantly higher at the 1-year study than at the baseline study [mean 104.0 (standard error 9.9) vs 92.3 (8.4) ml min$^{-1}$ 1.73 m$^{-2}$, $n$ = 13, $P$ = 0.04], with a similar but statistically insignificant trend at the 2-year study ($P$ = 0.19) (Figure 1). Although some individual children showed either progression or partial recovery of tubular toxicity during the interval from the baseline to the 2-year study, no statistically significant changes were observed in the group as a whole.

© Cancer Research Campaign 1998

**Figure 1** Changes in glomerular filtration rate (GFR) in individual patients with time after completion of cisplatin treatment. The lower limit of normal is shown

### DISCUSSION

The results of this follow-up study confirm previous reports of the frequency, nature and severity of cisplatin-induced glomerular and proximal nephron nephrotoxicity in children, leading to clinically important reductions in GFR and serum magnesium concentration (Ettinger et al, 1981; Hayes et al, 1981; Womer et al, 1985; Goren et al, 1986; Sheldon et al, 1987; Bianchetti et al, 1990; Brock et al, 1991). These adverse effects occurred despite the use of hyper-hydration protocols, which appear to reduce the frequency and severity of nephrotoxicity, rather than abolish it altogether (Daugaard and Abildgaard, 1989). Although few children suffered from clinically significant toxicity, acute and/or chronic renal failure in 3 out of 35 patients led to considerable constraints in the choice of further chemotherapy. Despite the frequency of chronic hypomagnesaemia, there were no episodes of paraesthesia, tetany or convulsions after completion of treatment in this study, although such complications are well documented (Bellin and Selim, 1988).

A total of 58% of evaluable children had a GFR below the lower limit of the reference range, and 29% were hypomagnesaemic. These two major features of cisplatin nephrotoxicity did not appear to be necessarily associated in individual patients. Although the only four children with serum magnesium concentrations <0.60 mmol $l^{-1}$ were among the 22 who had received >500 mg $m^{-2}$ cisplatin, the dose rate at which cisplatin was given in each course appeared to be the major risk factor for the development of toxicity. Patients who had received cisplatin at a 'low' rate of 40 mg $m^{-2}$ $day^{-1}$ suffered less severe and frequent glomerular and proximal nephron toxicity than those who had received a 'higher' rate. Glomerular, but not tubular, toxicity appeared to be partially reversible with time after completion of cisplatin.

Of nine hypomagnesaemic children, two had unequivocal hypermagnesuria, and two had a fractional excretion of magnesium in the upper half of the reference range. This implies inappropriate magnesuria due to proximal nephron damage, particularly in the thick ascending limb of the loop of Henle, as the normal renal response to hypomagnesaemia leads to a reduced fractional excretion (Shils, 1969; Dirks, 1983; Suki and Rouse, 1991).

Evidence of impaired proximal nephron reabsorption of other electrolytes and small molecules was observed, with glycosuria in 72% of children, mild hyponatraemia in 26% and hypocalcaemia in 14%, as described previously (Lammers et al, 1984; Vassal et al, 1987). Hypocalciuria despite normal or slightly high plasma calcium concentrations has been reported (Bianchetti et al, 1990), occurring alone or with hypomagnesaemia and hypokalaemic metabolic alkalosis, suggesting a distal nephron lesion. Of 28 evaluable children in this study, six had a fractional excretion of calcium at the lower limit of the normal range. Two were also hypomagnesaemic, but none were hypokalaemic or alkalotic.

The frequent occurrence of subclinical distal nephron toxicity was suggested by the failure to achieve adequate urine pH or osmolality in the majority of children, but these abnormalities are relatively non-specific. Dynamic functional investigations such as acid loading and fluid deprivation studies were not performed because of their unpleasant or potentially dangerous nature (Skinner et al, 1991). However, no child had acidaemia or symptoms suggesting significant impairment of urinary concentration.

Clearer evidence of subclinical tubular damage was provided by the frequency of raised urinary loss of low-molecular-weight proteins and of renal tubular enzymes.

It is not clear why only some patients receiving cisplatin develop severe glomerular impairment. Considerable interindividual differences in toxicity were apparent in this study, even between patients of similar age and receiving similar administration schedules and total doses of cisplatin. It is possible that interindividual variability in cisplatin pharmacokinetics may be at least partly responsible (Reece et al, 1987).

This study evaluated the importance of the dose rate of administration of cisplatin during treatment courses, quantified in mg $m^{-2}$ $day^{-1}$ of cisplatin treatment (for example during a 5-day cisplatin course). This is not 'dose intensity', which is generally measured per day of overall treatment duration (for example 18 weeks). The greater nephrotoxicity of higher dose rates of cisplatin has not been documented clearly before in children but is unsurprising as it is generally considered that the renal toxicity of cisplatin is reduced by prolonged infusion. The results of this study suggest that cisplatin dose rates of 40 mg $m^{-2}$ $day^{-1}$ should be used in preference to higher dose rates in children. The lack of a clear correlation between total cisplatin dose and the severity of nephrotoxicity is consistent with other detailed long-term follow-up studies in children (Womer et al, 1985; Sheldon et al, 1987; Brock et al, 1991), but contrasts with some earlier studies (Pratt et al, 1981; Sexauer et al, 1985; Goren et al, 1986). It is possible that children receiving higher total doses are at greater risk of significant toxicity, but that this effect is obscured by interindividual variability in susceptibility. Similarly, the lack of obvious influence of other potentially nephrotoxic treatment is in agreement with most previous reports (Womer et al, 1985; Sheldon et al, 1987; Brock et al, 1991), although effects in individual children can not be excluded, and may have contributed to the occurrence of acute renal failure in two children in this study.

The apparent partial reversibility in glomerular impairment over the period of study is consistent with the observation of Brock (Brock et al, 1991), and is an important finding in view of the continued use of cisplatin in many combination chemotherapy protocols in children. Although 12 of the baseline studies were performed later than 3 months post-cisplatin, only six of these were included in the comparison of results from the baseline study with those from the 1-year study, with an interval between the baseline and 1-year study ranging from 5 to 8 months. This shorter period of follow-up is unlikely to have led to a misleading result, except perhaps by reducing the likelihood of observing any change in toxicity with time after treatment. Further investigations are continuing in a larger cohort of patients to enable clarification of the outcome up to 10 years after treatment. Although the long-term implications of chronic cisplatin-induced renal damage are unknown, the possibility that they may include hypertension or chronic renal failure is worrying.

In conclusion, this study has demonstrated the frequency of clinically important glomerular and proximal nephron damage in children treated with cisplatin, and has provided preliminary evidence that such toxicity is more severe in children receiving cisplatin dose rates greater than 40 mg $m^{-2}$ $day^{-1}$. Although both drugs cause glomerular damage, the nature of tubular damage after cisplatin is clearly distinct from that caused by ifosfamide (Skinner et al, 1993). There was no evidence of further deterioration in renal function after completion of cisplatin treatment, but the potential future implications for renal function in long-term survivors are a cause of concern.

### ACKNOWLEDGEMENTS

We are grateful to the Departments of Medical Physics and Clinical Biochemistry at the Royal Victoria Infirmary, Newcastle upon Tyne for their assistance with the investigations; and to Dr AW Skillen for the retinol-binding protein and renal tubular enzyme assays. Dr R Skinner was an MRC Training Fellow. We also thank the Special Trustees of Newcastle Health Authority and the North of England Children's Cancer Research Fund for additional financial support.

### REFERENCES

Bajorin DF, Sarosdy MF, Pfister DG, Mazumdar M, Motzer RJ, Scher HI, Geller NL, Fair WR, Herr H, Sogani P, Sheinfeld J, Ruso P, Vlamis V, Carey R, Vogelzang NJ, Crawford ED and Bosl GJ (1993) Randomized trial of etoposide and cisplatin versus etoposide and carboplatin in patients with good risk germ cell tumors: a multiinstitutional study. *J Clin Oncol* **11**: 598–606

Bellin SL and Selim M (1988) Cisplatin-induced hypomagnesemia with seizures: a case report and review of the literature. *Gynecol Oncol* **30**: 104–113

Bianchetti MG, Kanaka C, Ridolfi-Luthy A, Wagner HP, Hirt A, Paunier L, Peheim E and Oetliker OH (1990) Chronic renal magnesium loss, hypocalciuria and mild hypokalaemic metabolic alkalosis after cisplatin. *Pediatr Nephrol* **4**: 219–222

Brock PR, Koliouskas DE, Barratt TM, Yeomans E and Pritchard J (1991) Partial reversibility of cisplatin nephrotoxicity in children. *J Pediatr* **118**: 531–534

Daugaard G and Abildgaard U (1989) Cisplatin nephrotoxicity. *Cancer Chemother Pharmacol* **25**: 1–9

Dirks JH (1983) The kidney and magnesium regulation. *Kidney Int* **23**: 771–777

Ettinger LJ, Douglass HO, Higby DJ, Mindell ER, Nime F, Ghoorah J and Freeman AI (1981) Adjuvant adriamycin and cis-diamminedichloroplatinum (cis-platinum) in primary osteosarcoma. *Cancer* **47**: 248–254.

Goren MP, Wright RK and Horowitz ME (1986) Cumulative renal tubular damage associated with cisplatin nephrotoxicity. *Cancer Chemother Pharmacol* **18**: 69–73

Hayes FA, Green AA, Casper J, Cornet J and Evans WE (1981) Clinical evaluation of sequentially scheduled cisplatin and VM26 in neuroblastoma: response and toxicity. *Cancer* **48**: 1715–1718

Lammers PJ, White L and Ettinger LJ (1984) Cis-platinum-induced renal sodium wasting. *Med Pediatr Oncol* **12**: 343–346

Madias NE and Harrington JT (1978) Platinum nephrotoxicity. *Am J Med* **65**: 307–314

Morris Jones PH and Craft AW (1990) Childhood cancer: cure at what cost? *Arch Dis Child* **65**: 638–640

Pratt CB, Hayes A, Green AA, Evans WE, Senzer N, Howarth CB, Ransom JL and Crom W (1981) Pharmacokinetic evaluation of cisplatin in children with malignant solid tumors: a phase II study. *Cancer Treat Rep* **65**: 1021–1026

Reece PA, Stafford I, Russell J, Khan M and Gill PG (1987) Creatinine clearance as a predictor of ultrafilterable platinum disposition in cancer patients treated with cisplatin: relationship between peak ultrafilterable platinum plasma levels and nephrotoxicity. *J Clin Oncol* **5**: 304–309

Schilsky RL, Barbock A and Ozols RF (1982) Persistent hypomagnesemia following cisplatin chemotherapy for testicular cancer. *Cancer Treat Rep* **66**: 1767–1769

Sexauer CL, Khan A, Burger PC, Krischer JP, van Eys J, Vats T and Ragab AH (1985) Cisplatin in recurrent pediatric brain tumors. A POG phase II study. *Cancer* **56**: 1497–1501

Sheldon W, Welch RJ, Bonham JR, Pearson ADJ and Craft AW (1987) Hypomagnesaemia following treatment of childhood cancer with cisplatinum. *Ann Clin Biochem* **4** (suppl.1): S85–S86

Shils ME (1969) Experimental human magnesium depletion. *Medicine* **48**: 61–85

Skinner R, Pearson ADJ, Coulthard MG, Skillen AW, Hodson AW, Goldfinch ME, Gibb I and Craft AW (1991) Assessment of chemotherapy-associated nephrotoxicity in children with cancer. *Cancer Chemother Pharmacol* **28**: 81–92

Skinner R, Sharkey IM, Pearson ADJ and Craft AW (1993) Ifosfamide, mesna and nephrotoxicity in children. *J Clin Oncol* **11**: 173–190

Suki WN and Rouse D (1991) Renal transport of calcium, magnesium, and phosphorus. In *The Kidney*, Brenner BM and Rector FC, Jr (eds), Vol. I. pp. 380–423. WB Saunders: Philadelphia

Vassal G, Rubie H, Kalifa C, Hartmann O and Lemerle J (1987) Hyponatremia and renal sodium wasting in patients receiving cisplatinum. *Pediatr Hematol Oncol* **4**: 337–344

Womer RB, Pritchard J and Barratt TM (1985) Renal toxicity of cisplatin in children. *J Pediatr* **106**: 659–663

© Cancer Research Campaign 1998


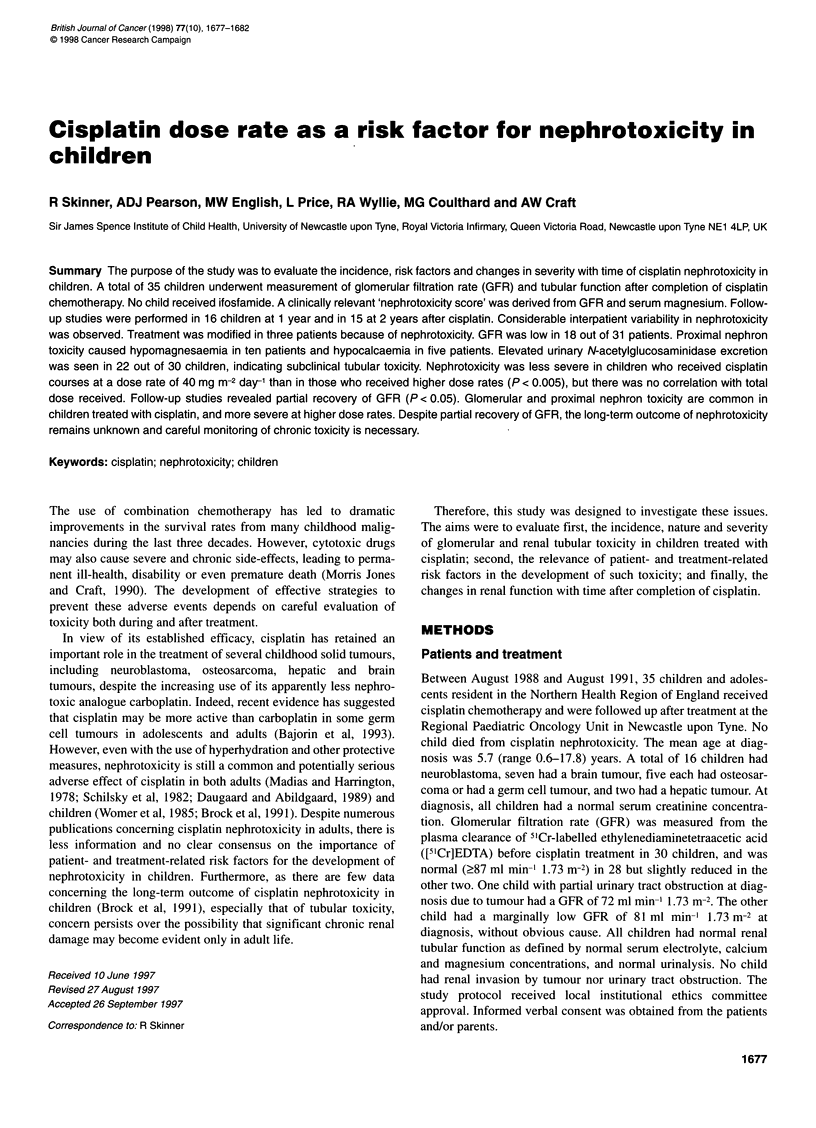

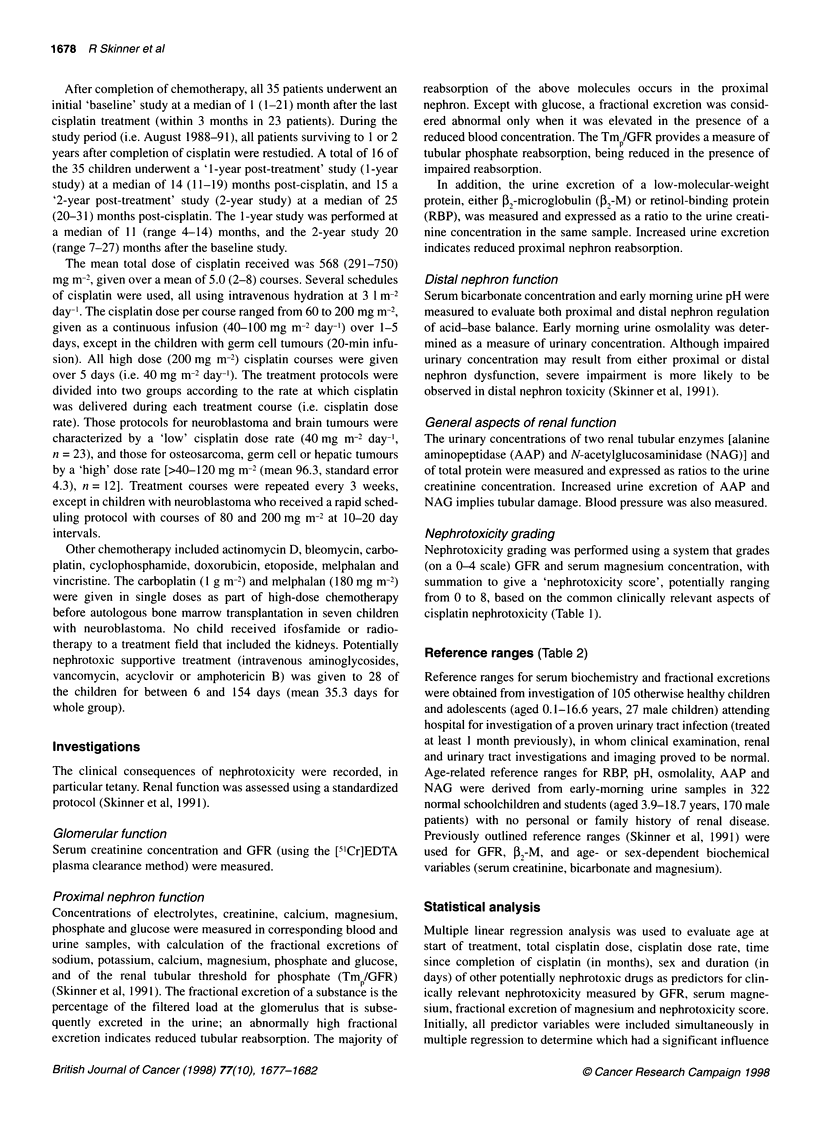

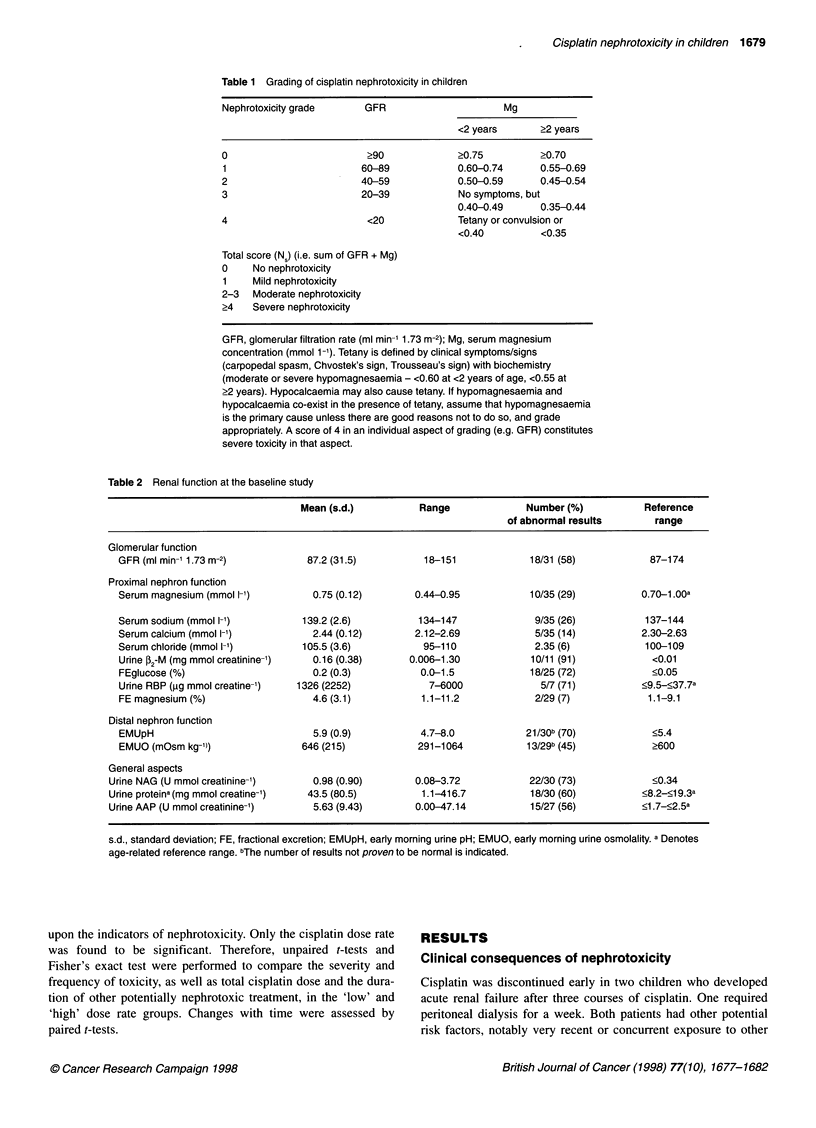

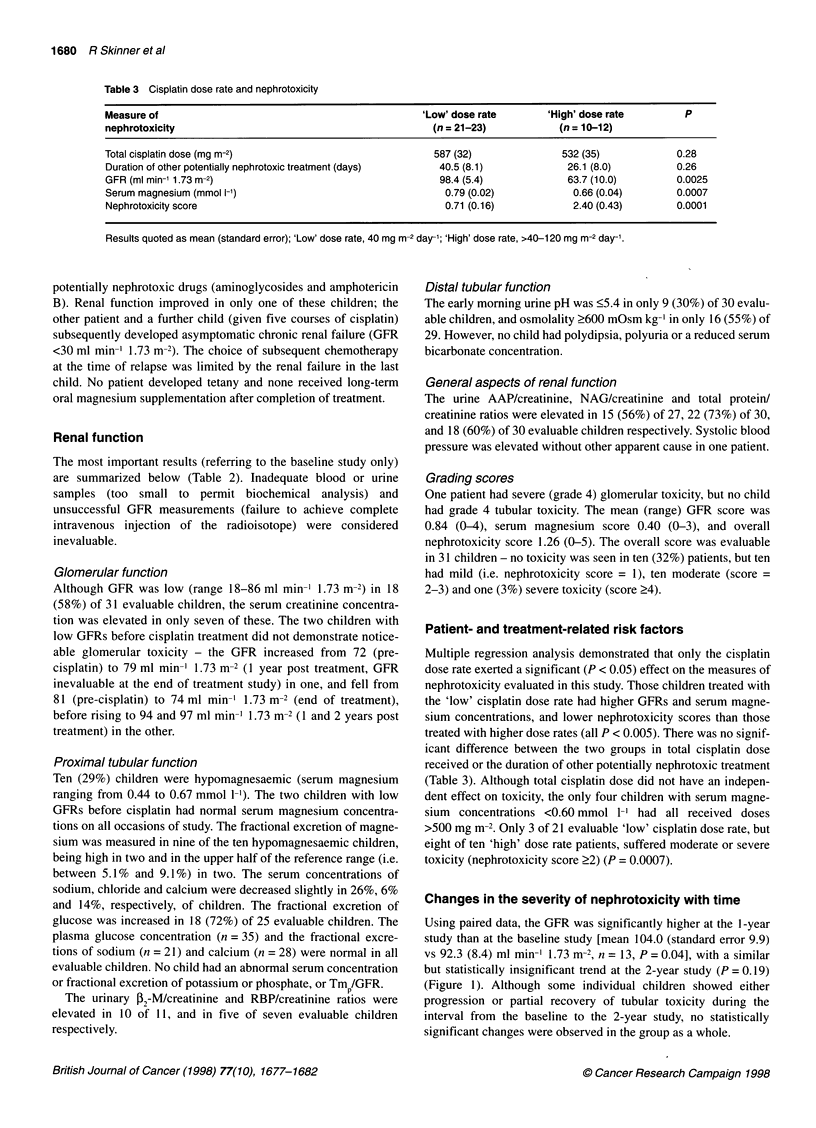

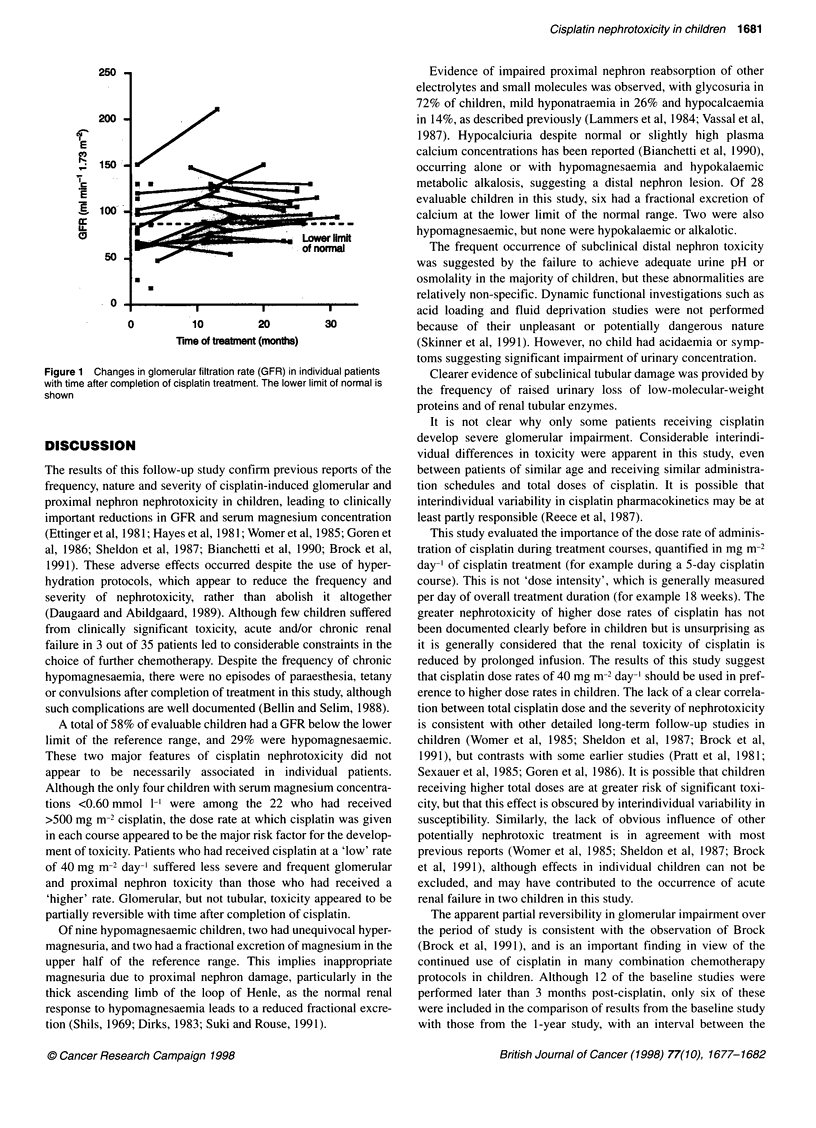

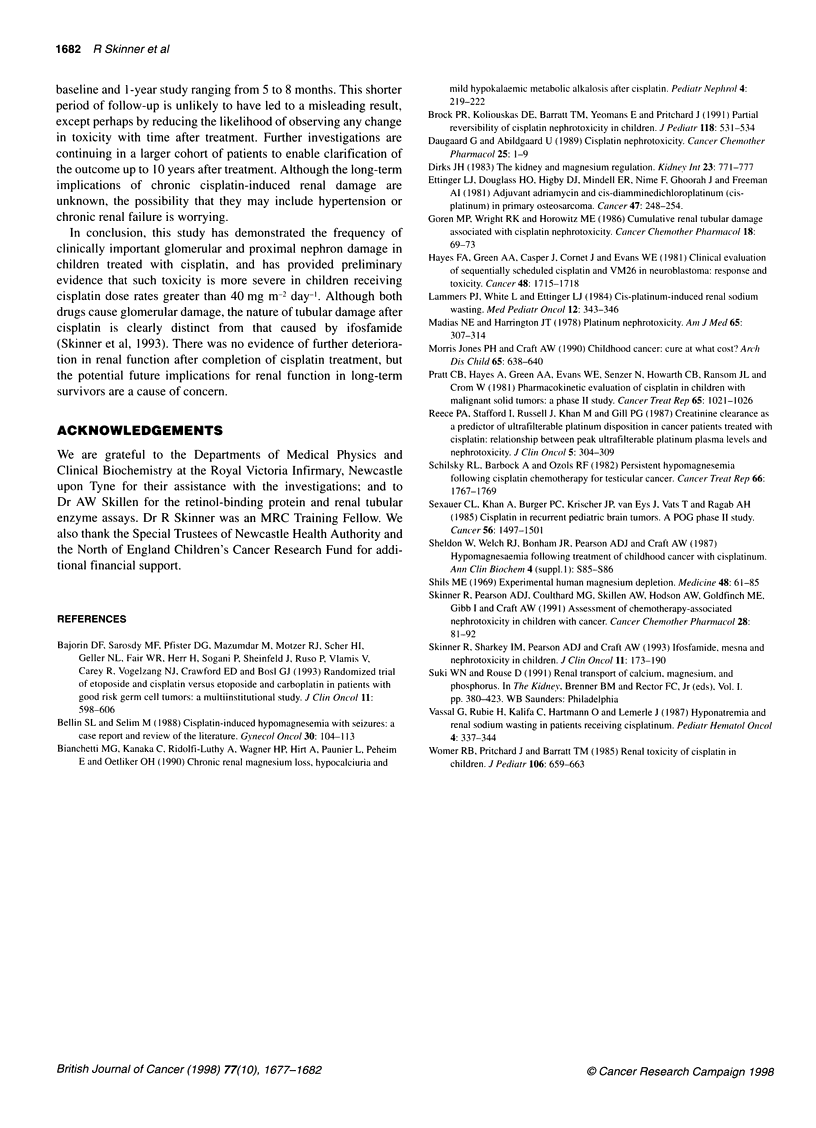


## References

[OCR_00630] Bajorin D. F., Sarosdy M. F., Pfister D. G., Mazumdar M., Motzer R. J., Scher H. I., Geller N. L., Fair W. R., Herr H., Sogani P. (1993). Randomized trial of etoposide and cisplatin versus etoposide and carboplatin in patients with good-risk germ cell tumors: a multiinstitutional study.. J Clin Oncol.

[OCR_00639] Bellin S. L., Selim M. (1988). Cisplatin-induced hypomagnesemia with seizures: a case report and review of the literature.. Gynecol Oncol.

[OCR_00643] Bianchetti M. G., Kanaka C., Ridolfi-Lüthy A., Wagner H. P., Hirt A., Paunier L., Peheim E., Oetliker O. H. (1990). Chronic renal magnesium loss, hypocalciuria and mild hypokalaemic metabolic alkalosis after cisplatin.. Pediatr Nephrol.

[OCR_00650] Brock P. R., Koliouskas D. E., Barratt T. M., Yeomans E., Pritchard J. (1991). Partial reversibility of cisplatin nephrotoxicity in children.. J Pediatr.

[OCR_00658] Dirks J. H. (1983). The kidney and magnesium regulation.. Kidney Int.

[OCR_00660] Ettinger L. J., Douglass H. O., Higby D. J., Mindell E. R., Nime F., Ghoorah J., Freeman A. I. (1981). Adjuvant adriamycin and cis-diamminedichloroplatinum (cis-platinum) in primary osteosarcoma.. Cancer.

[OCR_00665] Goren M. P., Wright R. K., Horowitz M. E. (1986). Cumulative renal tubular damage associated with cisplatin nephrotoxicity.. Cancer Chemother Pharmacol.

[OCR_00670] Hayes F. A., Green A. A., Casper J., Cornet J., Evans W. E. (1981). Clinical evaluation of sequentially scheduled cisplatin and VM26 in neuroblastoma: response and toxicity.. Cancer.

[OCR_00675] Lammers P. J., White L., Ettinger L. J. (1984). Cis-platinum-induced renal sodium wasting.. Med Pediatr Oncol.

[OCR_00679] Madias N. E., Harrington J. T. (1978). Platinum nephrotoxicity.. Am J Med.

[OCR_00683] Morris-Jones P. H., Craft A. W. (1990). Childhood cancer: cure at what cost?. Arch Dis Child.

[OCR_00687] Pratt C. B., Hayes A., Green A. A., Evans W. E., Senzer N., Howarth C. B., Ransom J. L., Crom W. (1981). Pharmacokinetic evaluation of cisplatin in children with malignant solid tumors: a phase II study.. Cancer Treat Rep.

[OCR_00693] Reece P. A., Stafford I., Russell J., Khan M., Gill P. G. (1987). Creatinine clearance as a predictor of ultrafilterable platinum disposition in cancer patients treated with cisplatin: relationship between peak ultrafilterable platinum plasma levels and nephrotoxicity.. J Clin Oncol.

[OCR_00699] Schilsky R. L., Barlock A., Ozols R. F. (1982). Persistent hypomagnesemia following cisplatin chemotherapy for testicular cancer.. Cancer Treat Rep.

[OCR_00704] Sexauer C. L., Khan A., Burger P. C., Krischer J. P., van Eys J., Vats T., Ragab A. H. (1985). Cisplatin in recurrent pediatric brain tumors. A POG Phase II study. A Pediatric Oncology Group Study.. Cancer.

[OCR_00714] Shils M. E. (1969). Experimental human magnesium depletion.. Medicine (Baltimore).

[OCR_00716] Skinner R., Pearson A. D., Coulthard M. G., Skillen A. W., Hodson A. W., Goldfinch M. E., Gibb I., Craft A. W. (1991). Assessment of chemotherapy-associated nephrotoxicity in children with cancer.. Cancer Chemother Pharmacol.

[OCR_00723] Skinner R., Sharkey I. M., Pearson A. D., Craft A. W. (1993). Ifosfamide, mesna, and nephrotoxicity in children.. J Clin Oncol.

[OCR_00732] Vassal G., Rubie H., Kalifa C., Hartmann O., Lemerle J. (1987). Hyponatremia and renal sodium wasting in patients receiving cisplatinum.. Pediatr Hematol Oncol.

[OCR_00737] Womer R. B., Pritchard J., Barratt T. M. (1985). Renal toxicity of cisplatin in children.. J Pediatr.

